# The SyBil-AA real-time fMRI neurofeedback study: protocol of a single-blind randomized controlled trial in alcohol use disorder

**DOI:** 10.1186/s12888-018-1604-3

**Published:** 2018-01-17

**Authors:** Martin Fungisai Gerchen, Martina Kirsch, Nathalie Bahs, Patrick Halli, Sarah Gerhardt, Axel Schäfer, Wolfgang H. Sommer, Falk Kiefer, Peter Kirsch

**Affiliations:** 1 Department of Clinical Psychology, Central Institute of Mental Health (ZI), University of Heidelberg/Medical Faculty Mannheim, J5, 68159 Mannheim, Germany; 2 Department of Addiction Behavior and Addiction Medicine, Central Institute of Mental Health, University of Heidelberg/Medical Faculty Mannheim, Mannheim, Germany; 3 Department of Psychiatry and Psychotherapy, Central Institute of Mental Health, University of Heidelberg/Medical Faculty Mannheim, Mannheim, Germany; 4 Department of Psychopharmacology, Central Institute of Mental Health, University of Heidelberg/Medical Faculty Mannheim, Mannheim, Germany; 5 Bernstein Center for Computational Neuroscience Heidelberg/Mannheim, Mannheim, Germany

**Keywords:** Alcohol dependence, Addiction, Cue-reactivity, Ventral striatum, Inferior frontal gyrus, Functional magnetic resonance imaging, Brain-computer interface

## Abstract

**Background:**

Alcohol Use Disorder is a highly prevalent mental disorder which puts a severe burden on individuals, families, and society. The treatment of Alcohol Use Disorder is challenging and novel and innovative treatment approaches are needed to expand treatment options. A promising neuroscience-based intervention method that allows targeting cortical as well as subcortical brain processes is real-time functional magnetic resonance imaging neurofeedback. However, the efficacy of this technique as an add-on treatment of Alcohol Use Disorder in a clinical setting is hitherto unclear and will be assessed in the Systems Biology of Alcohol Addiction (SyBil-AA) neurofeedback study.

**Methods:**

*N* = 100 patients with Alcohol Use Disorder will be randomized to 5 parallel groups in a single-blind fashion and receive real-time functional magnetic resonance imaging neurofeedback while they are presented pictures of alcoholic beverages. The groups will either downregulate the ventral striatum, upregulate the right inferior frontal gyrus, negatively modulate the connectivity between these regions, upregulate, or downregulate the auditory cortex as a control region. After receiving 3 sessions of neurofeedback training within a maximum of 2 weeks, participants will be followed up monthly for a period of 3 months and relapse rates will be assessed as the primary outcome measure.

**Discussion:**

The results of this study will provide insights into the efficacy of real-time functional magnetic resonance imaging neurofeedback training in the treatment of Alcohol Use Disorder as well as in the involved brain systems. This might help to identify predictors of successful neurofeedback treatment which could potentially be useful in developing personalized treatment approaches.

**Trial registration:**

The study was retrospectively registered in the German Clinical Trials Register (trial identifier: DRKS00010253; WHO Universal Trial Number (UTN): U1111–1181-4218) on May 10th, 2016.

## Background

Alcohol use disorder (AUD) is characterized by drinking of alcohol despite negative or harmful consequences, a loss of control over drinking behavior, the development of craving for alcohol, and the occurrence of withdrawal symptoms. AUD puts a severe burden on individuals, families, and societies [1] and is highly prevalent in western countries with a twelve-month prevalence of 13.9% in the United States [2] and around 7% in Europe, where prevalence differs over a wide range between countries [3].

More than 80% of people suffering from AUD do not receive formal treatment [2], and of those who do, only 25–43% remain abstinent [4, 5]. To increase the efficacy of AUD treatment, novel treatment approaches are needed which would optimally be designed as modules that can flexibly be administered in personalized multimodal treatment programs.

The progress of basic neuroscientific knowledge about alcohol addiction and advances in methodology now make neuroscience-based treatment approaches like transcranial magnetic stimulation (TMS) [6–9], transcranial direct current stimulation (tDCS) [10–13], deep brain stimulation (DBS) [14, 15], and real-time functional magnetic resonance neurofeedback (rtfMRI NFB) [16–18] available, which are able to target disease-related brain regions and brain processes with more or less precision.

One process that has prominently been linked to AUD and is a promising aim for targeted neuroscience-based interventions is the cue-reactivity response [19–22], the reaction to the presentation of alcohol-related stimuli, e.g. pictures, odors, or taste of alcoholic beverages. In the brain, core regions associated with cue-reactivity are the ventral striatum (VS), the anterior cingulate cortex (ACC), and the ventromedial prefrontal cortex (vmPFC) [23, 24].

Reviewing the cognitive neuroscience literature on behavioral change in AUD, Naqvi & Morgenstern [25] concluded that enhanced reactivity of the ventral striatum to alcohol and alcohol-related cues together with impairments in prefrontal control regions are the major factors responsible for maintaining AUD. Along these lines, Becker et al. [26] have recently demonstrated enhanced striatal reward sensitivity and impairments in prefrontal-striatal connectivity in patients with AUD in a monetary reward task, hinting at a more generalized impairment in reward processing in AUD, which has also been found being predictive for neural changes during psychotherapeutic treatment [27].

To target the cortical and subcortical processes involved in cue-reactivity and cognitive control, real-time fMRI Neurofeedback is a promising approach. In rtfMRI NFB participants are shown a near real-time feedback signal indicating a specific brain process while they are lying in an MR scanner and are instructed to influence this feedback signal in a desired direction (see Fig. 2 for an illustration). In contrast to other treatment approaches like pharmacological interventions, TMS, or DBS, in rtfRMI NFB patients are actively engaging to influence a brain process, which might have additional psychological therapeutic benefits by promoting self-efficacy and self-regulation [28]. Furthermore, in comparison, rtfMRI NFB offers a relatively high spatial acuity, especially in subcortical brain regions.

While rtfRMI NFB treatment is a relatively new tool in psychiatry, first results are promising. Initial evidence has been presented that rtfRMI NFB has effects for example in major depressive disorder [29–34], schizophrenia [35–37], attention deficit hyperactivity disorder [38, 39], phobia [40], autism [41], posttraumatic stress disorder [42], and Borderline personality disorder [43].

With regard to substance use disorders, rtfMRI NFB has been mainly explored in the treatment of nicotine addiction [44]. Several studies have demonstrated a reduction of nicotine craving through NFB [45–48]. Interestingly, a recent study has found that including functional connectivity information in the feedback signal leads to a higher reduction of nicotine craving [49].

So far, only few studies have been conducted to assess the use of rtfRMI NFB as an interventional tool in AUD. Showing that neurofeedback training with feedback from individually chosen cortical regions was able to reduce craving in patients with AUD directly after neurofeedback training, Karch et al. [50] demonstrated the feasibility of neurofeedback interventions in AUD. In a study with non-treatment seeking heavy social drinkers, Kirsch et al. [18] found that participants who received real feedback in comparison to a yoked feedback group and a sham control group were able to learn to downregulate their ventral striatum activation during presentation of pictures of alcoholic beverages. They could further show that downregulation of the VS was correlated with activation in the right inferior frontal gyrus in the real feedback group, which is in accordance with the model of Naqvi & Morgenstern [25].

In the Systems Biology of Alcohol Addiction (SyBil-AA) rtfRMI NFB study we are now testing whether neurofeedback training will have beneficial effects in heavily impaired AUD patients in a clinical setting. The results of this study will provide insights into the efficacy of NFB training in AUD, the involved brain systems, and might help to identify predictors of treatment success which could potentially be useful in developing personalized treatment approaches.

## Methods/Design

**Fig. 1:**
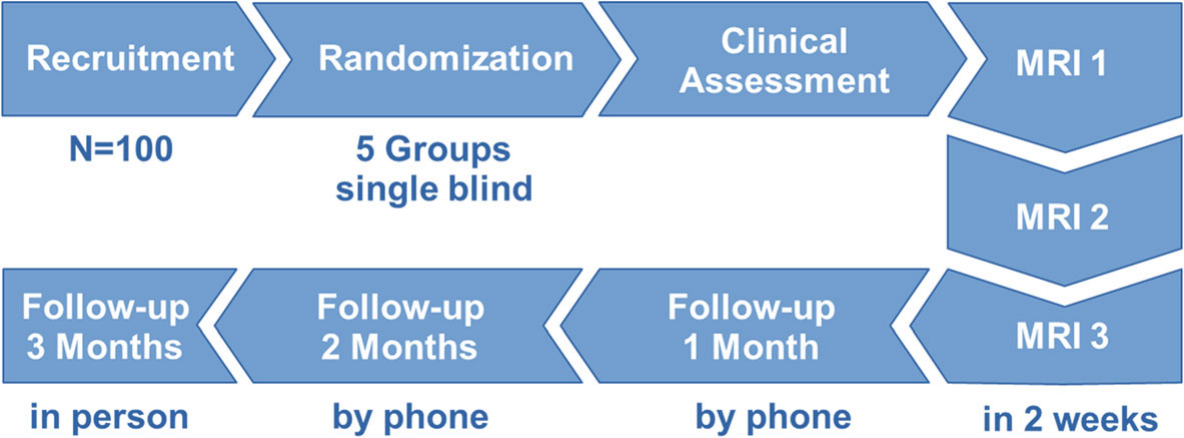
Study flow chart. *N* = 100 patients with a diagnosis of alcohol addiction from the local inpatient and outpatient addiction clinics are randomized to 5 single-blind groups, receive rtfMRI NFB treatment on 3 days within two weeks, and are followed up monthly for 3 months

To investigate the effect of rtfMRI NFB and identify the underlying neural mechanisms we will include *N* = 100 patients with a diagnosis of alcohol use disorder recruited from the outpatient and inpatient clinics of the Department of Addiction Behavior and Addiction Medicine at the Central Institute of Mental Health (CIMH), Mannheim, Germany. Participants will be randomly assigned to one of 5 parallel groups. All groups will receive true rtfMRI NFB from different brain processes and will be followed up monthly for three months (Fig. 1).

### Participant eligibility and recruitment

Eligible participants are between 18 and 65 years with normal or corrected-to-normal vision. They are diagnosed with alcohol dependence according to the International Classification of Diseases, tenth edition (ICD-10: F10.2) with controlled abstinence for at least 5 and a maximum of 21 days prior to study inclusion. Additionally, a medically supervised detoxification program (treatment of withdrawal symptoms with short-acting benzodiazepines or clomethiazole) has to have been completed for at least 3 days.

Exclusion criteria are: meeting the criteria of any axis I psychiatric disorder according to the Diagnostic and Statistical Manual of Mental Disorders, fourth edition (DSM-IV) and the ICD-10 within the past 12 months, with the exception of alcohol and nicotine abuse/dependence and a mild depression related to alcohol consumption or detoxification. Further exclusion criteria are a positive urine drug screening, current use of psychotropic or anticonvulsive medications, epilepsy or neurological or severe medical illness, suicidal tendencies, pregnancy, and breastfeeding.

For recruitment, participants are informed by a psychologist of the study team about the study purpose and are able to ask all questions concerning the study content. Participants are then asked to provide written informed consent before being screened for in- and exclusion criteria. Participants are able to withdraw their consent at any time.

### Screening assessment & group allocation

Screening for in- and exclusion criteria includes the Structured Clinical Interview for DSM-IV (SKID-I) [51] conducted by a clinical psychologist. Either during the detoxification program or the screening assessment a drug and pregnancy test is conducted. Please see Table 1 for further information on the schedule of measurements.

**Table 1:** Schedule of measurements conducted in the study

Measurement	S^a^	T0^a^	MRI1^a^	MRI2^a^	MRI3^a^	FU1/2^b^	FU3^a^
Sociodemographic information		X					
Structured Clinical Interview (SKID – I)	X						
Drinking Assessment Interview (Form 90)		X					
Blood sample (AUD-related markers)	X						X
Urine sample (pregnancy, drugs)	X						
Current medication	X					X^c^	X^c^
Current smoking behavior		X^c^	X^c^	X^c^	X^c^	X^c^	X^c^
Breath alcohol test							X
Current drug use						X^c^	X^c^
Incentive Conflict Task		X					
Clinical & Personality Questionnaires							
Edinburgh Inventory of Handedness	X						
Beck Depression Inventory		X					
State Trait Anxiety Inventory		X					
Behavioral Inhibition/Approach System		X					
Barratt Impulsiveness Scale		X					
Fagerstrøm Test of Nicotine Dependence		X					
Alcohol-Related Questionnaires							
Quick Drinking Assessment Interview (Form 90-AQ)						X	X
Alcohol Dependence Scale		X					
Alcohol Abstinence Self-Efficacy Scale		X			X		X
German Inventory of Drinking Situations		X					
Obsessive Compulsive Drinking Scale		X			X		X
Alcohol Urge Questionnaire		X			X		X
Craving-Automatized-Scale-Alcohol		X			X		X
MRI							
Anatomical image (MPRAGE)			X	X	X		
Resting state			X	X	X		
Neurofeedback			X	X	X		
Transfer run			X		X		
Craving (visual analog scale) pre & post scanning			X	X	X		
Perceived control over NFB (visual analog scale)			X	X	X		

*S* screening assessment, *T0* baseline assessment, *MRI* rtfMRI scanning days, *FU* monthly follow-up. ^a^face-to-face; ^b^via telephone; ^c^self-reported (if unclear)

Participants who meet inclusion criteria are randomly assigned to one of five groups: three experimental and two control groups, respectively. Each group has to fulfil different tasks with either downregulating the ventral striatum, upregulating the right inferior frontal gyrus (rIFG), increasing the connectivity (negative correlation) between rIFG and VS, downregulating the auditory cortex (downregulation control) or upregulating the auditory cortex (upregulation control). The auditory cortex was chosen as control region because it is not involved in cue-reactivity [22].

Group allocation of included participants is conducted by the study team with a computer-generated random list based on the sequence of inclusion. No stratification factors are used in the allocation process.

### Baseline assessment

After group allocation, all participants take part in a baseline assessment about demographic questions, alcohol use, actual medication, personality, and clinical symptoms (see Table 1). In addition, participants conduct the computer-based Incentive Conflict Task (ICT, [52]) during the baseline assessment.

### Neurofeedback setup

rtfMRI NFB is conducted at Siemens 3 T Tim Trio scanners (Siemens Healthineers, Erlangen, Germany) at the Central Institute of Mental Health in Mannheim, Germany. Each participant receives neurofeedback training on three scanning days within a period of two weeks. On each day a 5:21 min T1-weighted anatomical MPRAGE scan, a 12:00 min functional resting state scan with eyes closed, and three neurofeedback runs of 9:29 min are conducted. At the beginning of each session an MPRAGE image is acquired with a time of repetition (TR) of 2.3 s, an echo time (TE) of 3.03 ms, 9° flip angle, a field of view of 256 mm with 192 sagittal slice, a matrix size of 256 × 256 mm, 1x1x1 mm voxel size, and GRAPPA with iPAT = 2.

Resting state and NFB sessions are scanned with echo-planar imaging (EPI) sequences with a TR of 1.64 s, a TE of 30 ms, 73° flip angle, a field of view of 192 mm, 3x3x3 mm voxel size, 33 slices of 3 mm thickness with a distance factor of 33%, and GRAPPA with iPAT = 2.

**Fig. 2:**
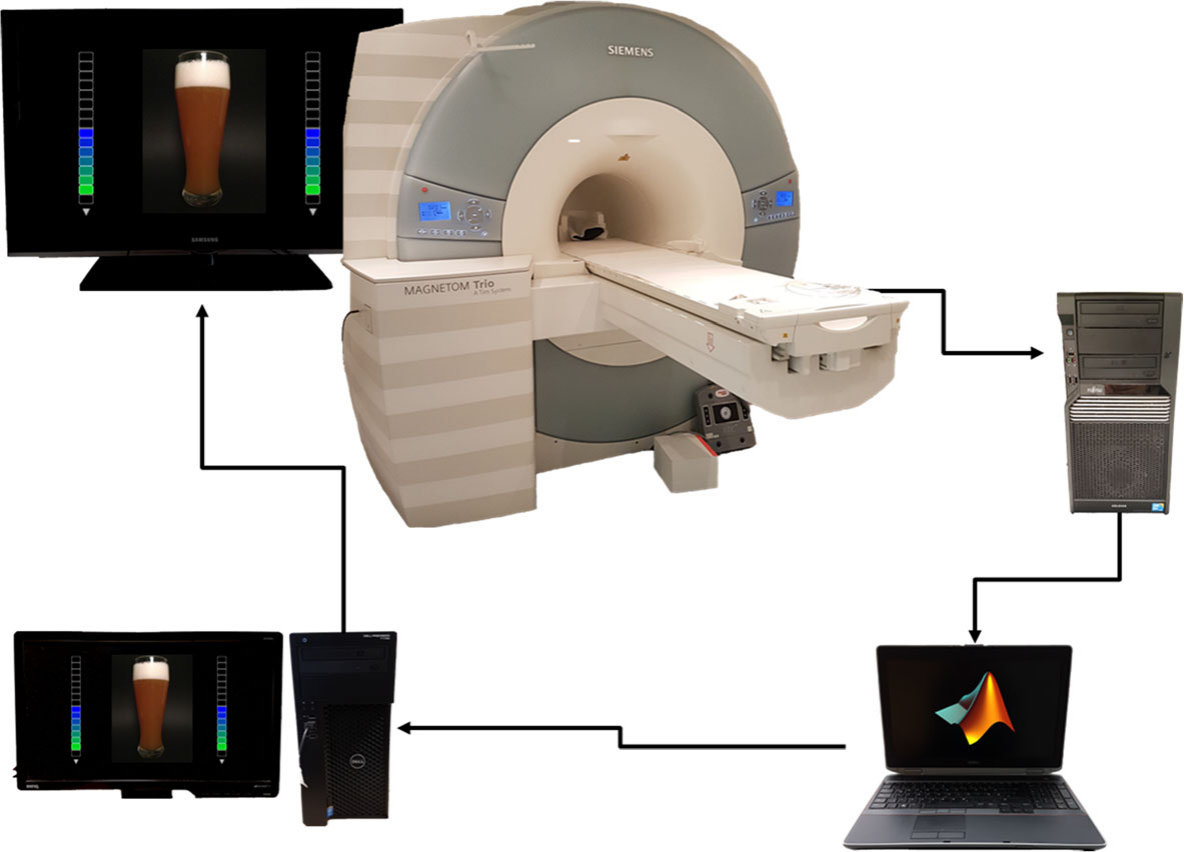
Real-time fMRI neurofeedback setup. Acquired images are reconstructed and send to a laptop running in-house MATLAB (MathWorks Inc., Sherborn, MA, USA) scripts based on SPM8 (Wellcome Department of Cognitive Neurology, London, UK) functions to preprocess the images and extract the neurofeedback signal. The feedback value is send to a computer running Presentation software (Neurobehavioral Systems, Inc., Albany, CA, USA) and presented to the participant in the scanner as a thermometer value besides a picture of an alcoholic beverage

During NFB scans, reconstructed DICOM images are transferred from the imaging computer to a laptop that preprocesses the images and extracts the neurofeedback signal by means of an in-house software. The feedback value is then send to a presentation computer and shown to the participant in the scanner as a thermometer value besides the picture of an alcoholic beverage preferred by the participant (beer, wine, or both). See Fig. 2 for an illustration. The thermometer is updated with every new volume. The order of pictures is randomized between participants.

rtfMRI NFB is conducted with in-house MATLAB (MathWorks Inc., Sherborn, MA, USA) scripts based on SPM8 functions (Wellcome Department of Cognitive Neurology, London, UK), and Presentation software (Neurobehavioral Systems, Inc., Albany, CA, USA) to present pictures and the feedback signal. On each scanning day, the acquired anatomical image is first segmented and normalized to MNI standard space. The inverse deformations of the normalization are then applied to warp the masks of the target regions into subject space.

During rtfMRI NFB scanning, functional images are realigned to the mean of the first 10 functional images and resliced. Then, the mask images are resampled to the space of the current image and mean intensity values from the voxels in the target regions are extracted.

For activation NFB, intensity values during presentation of alcohol images are averaged over the last three volumes (moving average) to stabilize the feedback signal and percent signal change with regard to the preceding fixation cross block is calculated. The scale of the feedback thermometer is adaptively adjusted to the maximum absolute signal change and the feedback signal is presented to the participant.

For connectivity NFB, partial correlations between the intensity values of the VS and right IFG adjusted for the cerebro-spinal fluid signal over the last 15 volumes are used to calculate the feedback signal. Again, the scale of the feedback thermometer is adaptively adjusted to the maximum absolute value reached. In this exploratory group, the goal of the intervention is to increase the inhibiting influence of the rIFG to the VS and the participants are instructed to downregulate the depicted process. However, a negative correlation between VS and rIFG could arise from the desired state, but also from an inverse behavior of the system with increased VS and diminished frontal activity. To enforce the top-down inhibitory state of the system we included the additional constraints in the connectivity feedback that the VS should be downregulated and the rIFG should be upregulated and switch negative feedback values to positive if these constraints are not met.

### Task design

**Fig. 3:**
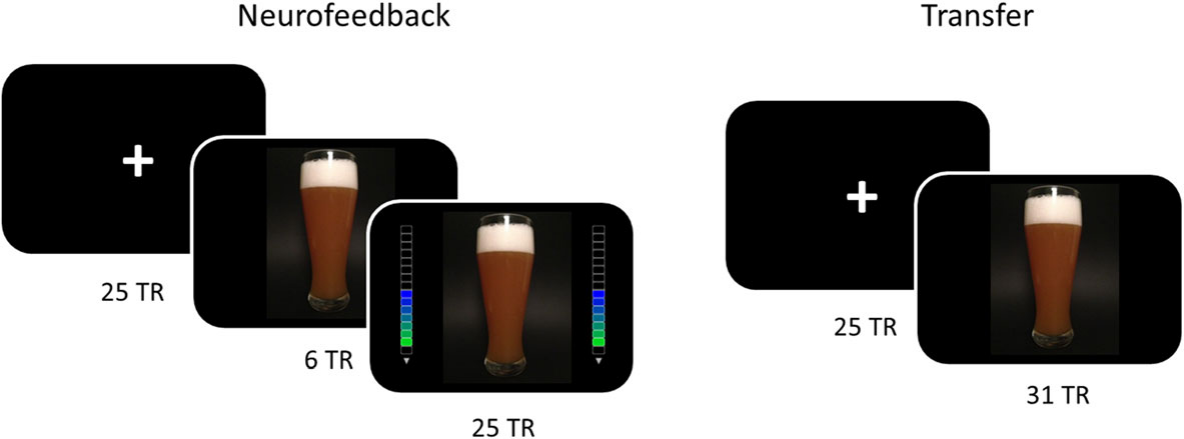
Experimental design. Design of neurofeedback (left) and transfer (right) tasks. Each run consists of 6 repetitions of the displayed sequences. The last (third) runs on the first and third scanning day are conducted as transfer sessions without the neurofeedback signal. TR: Time of Repetition (1 TR = 1.64 s)

NFB sessions have a block design with alternating presentation of a fixation cross (baseline; 41 s) and a picture of a preferred alcoholic beverage (beer, wine, or both; approx. 51 s), each for 6 times. Approximately 10 s after onset of the alcoholic pictures a feedback thermometer is shown on both sides of the picture and updated every TR (feedback; 41 s). On the first and third day, the last (third) run is conducted as a transfer run without presentation of the feedback signal (Fig. 3).

On each scanning day participants rate their craving on a visual analog scale directly before and after scanning. After completing the third MRI scanning session, participants are asked to answer alcohol-related questionnaires (see Table 1).

### Follow-up assessments

Follow-up assessments take place monthly after the last MRI session over a period of three months. At the first two time points participants are contacted by phone and asked about their current medication, their abuse of tobacco, nicotine and drugs and a potential alcohol relapse (see Table 1). Three months after the last MRI session, patients are invited to a final appointment at the CIMH. During this session, blood samples are collected and participants are asked about substance use and answer alcohol-related questionnaires (see Table 1). For the final appointment, patients receive a monetary compensation of 50 €.

### Statistical analyses

As primary outcome analysis we will conduct group comparisons of relapse rates with survival analysis and assess reduction of alcohol intake during the follow-up phase. As secondary and exploratory outcome measures we will assess whether rtfMRI NFB reduces alcohol craving during the NFB sessions, how brain activation and brain connectivity changes within and between the sessions, whether functional or structural MRI markers can be identified that predict neurofeedback learning and the success of the intervention, and whether differences in efficacy of the group-specific interventions do exist.

To analyze the effects of rtfMRI NFB, offline data analysis will be conducted with MATLAB and SPM. Data preprocessing will consist of slice-time correction, realignment, segmentation of the anatomical image, normalization to the MNI space, smoothing, and identification of outlier volumes with excessive head motion. The preprocessed data will be used in first level single subject analyses in which the time courses of the experimental conditions will be convolved with the canonical hemodynamic response function and used in a general linear model to estimate brain responses during rtfMRI NFB. Then, first level results will be used in second level group comparisons to estimate differential activation between groups, test for associations with clinical variables, and assess the predictive value of imaging markers for the future course of the disease. Additionally, large-scale adaptations in functional networks during rtfMRI NFB will be assessed by whole-brain psychophysiological interaction analysis [53].

Depending on the type of the data, missing data will either be left out from the analyses (e.g. fMRI data), or imputed (e.g. missing questionnaire items).

### Power calculations

Power calculations were conducted with the G*Power software package [54]. In the clinical analysis (follow-up measures) the power for detecting group differences at *p* < .05 under the assumption of a medium effect size (Cohen’s d = .5) is 78% with two sample *t*-tests when treatment (*n* = 60) and control groups (*n* = 40) are pooled. Assuming an effect size of f = .61 as observed for the neurofeedback effect on VS reduction in our pilot study [18], the neurofeedback analyses based on an ANOVA repeated measures model with a group x time contrast has a power of 89% in the VS and of 88% in the rIFG to detect FWE (Bonferroni) corrected significant results in the respective ROI analyses.

### Data management and dissemination

To ensure data quality, questionnaires are automatically scanned and entered into the study data base. Data management and monitoring is conducted by the study team. Study data will be stored on servers of the Central Institute of Mental Health separated from personal information of the participants. At the end of the study personal information will be deleted. The procedures comply with German data privacy laws. We will conduct interim analyses on the acquired data to present the study to scientific audiences (e.g. at conferences, meetings, etc.) during the data acquisition phase. Results of the final analyses will be published in scientific journals and presented on scientific conferences. Authorship will be defined in accordance to the German Research Foundation’s recommendations for safeguarding good scientific practice [55]. Study conduct is reported and audited in interim and final reports of the SyBil-AA consortium to the funding agency.

## Discussion

In the Systems Biology of Alcohol Addiction (SyBil-AA) rtfRMI NFB study we are assessing the efficacy of rtfRMI NFB on ventral striatal cue-reactivity and frontal control processes in the treatment of alcohol use disorder. To the best of our knowledge, the study is one of two ongoing clinical trials of rtfMRI NFB in AUD (see Cox et al. [56] for a description of the other trial), and will contribute to the understanding of the use of rtfMRI NFB as a clinical application in the context of a clinical treatment program for AUD.

Besides the primary goal of testing clinical effects, the study will provide data which will be useful to investigate how rtfMRI NFB is leading to changes in the brain, whether intervention success can be predicted by means of neurobiological signatures and clinical variables, and for which patients rtfMRI might be beneficial. Since we focus on ventral striatal activation to alcohol cues and its prefrontal control, it could be expected that particularly those patients will profit from the intervention that show substantial ventral striatal cue reactivity to alcohol cues prior to the intervention as it has been shown for other treatments before [57]. However, it has been shown that AUD is often associated with a shift of cue reactivity from ventral to dorsal striatum [58]. For those patients, showing a more habit like addiction behavior, instead of the ventral striatum, the dorsal striatum might be an interesting target for NFB interventions in AUD. Nevertheless, there is no doubt, that prefrontal control regions play a central role in craving and relapse [25]. Therefore, it could be speculated that patients receiving a PFC upregulation feedback might benefit from the NF treatment even if they do not show strong ventral but increased dorsal striatal activation to alcohol related cues.

An innovative condition in the present study is the connectivity feedback condition. It has been shown in the context of nicotine addiction that adding connectivity information to the feedback signal increases efficiency [49] and reduces cigarette craving but this has neither been shown for alcohol patients nor with respect a long-term outcome of a treatment.

However, the results from Kirsch et al. [18] could also imply that a critical factor for the treatment effect could be whether patients get the impression that they are able to actively control their own brain response to alcohol cues. Since patients can assume that the intention of the training is the reduction of alcohol cue associated craving, they might apply strategies to reduce this craving and, accordingly, to downregulate the feedback signal. Kirsch et al. [18] showed that successful VS downregulation was accompanied by the application of specific cognitive strategies, which might have let to the concurrent increase of prefrontal activation, although this activation was not fed back to the participants. Thus, the direction of the signal modulation could be a critical point in NFB, and patients receiving the downregulation instruction might show more effects than those receiving the upregulation instruction.

Future studies could further elaborate the effect of instruction by converting the feedback signal in upregulation conditions and instructing all participants to downregulate the signal, which from our experience seems to be the natural direction expected by patients in a cue-reactivity addiction context. This would also allow blinding the patients completely, not only with respect to the group (experimental vs. control) but also the kind of regulation they are expected to apply.

The study could also reveal additional mechanistic information about the mode of action of rtFMRI NF. Importantly, we conduct fMRI scans with whole-brain coverage, which will allow us to investigate the role of other brain regions like amygdala, insula cortex, or ventromedial PFC which were not directly targeted with NFB in our study but play important roles alcohol-related cue-reactivity [23, 24].

Another interesting aspect which might have an influence on the results is the type of control for neurofeedback (see e.g. [59]). We chose an active type of control in the NFB study in which participants from the control group receive a real feedback signal from a control region, which in our case is the auditory cortex that is not involved in cue-reactivity [22]. Other possibilities would be to use a control group without feedback, a group that just views feedback signal of other participants without instructions, or a yoke-control group that is told to regulate the process but receives feedback from another person. For task comparability we decided against the first two options, and for ethical reasons a yoke-control group in which participants would receive fake instructions is questionable. Our active-control groups will lead to results based on specific target brain processes, and not per se on brain regulation through NFB. However, differences between groups might still be related to differences in regulation difficulty.

There are some shortcomings of the protocol. First, the study is only single-blind. The staff members must prepare the individual feedback set up (definition of ROI, definition of direction) and are therefore not blinded. In future studies, a double-blind protocol could be implemented by separating staff that prepares the individual NF setup and staff that instructs the participants. However, we think it is worth to test the general applicability of rtfMRI NF to AUD patients before setting up such an extensive study protocol. Second, the study was retrospectively registered in the German Clinical Trials Register (DRKS00010253; WHO Universal Trial Number (UTN): U1111–1181-4218). Due to fast inclusion of the first patient, registration was conducted shortly after the begin of the study, but before any data was processed or analyzed (Inclusion of first patient: 03/30/2016; application for registration: 04/26/2016; registration date: 05/10/2016). However, the registration complies with the goals of the study described in the grant proposal and the study protocol will not be altered during the study. Finally, we have not defined stopping criteria, because we do not expect side effects besides those that can be expected from fMRI scanning, which is a safe and well-established technique. However, the principle investigators will decide whether to continue or terminate the study or specific parts of it if side effects were reported by the participants.

Overall, the study is well suited to provide valuable data on the efficacy of rtfMRI NFB as an add-on treatment for AUD in a clinical setting. Furthermore, the large amount of imaging data collected on the three scanning days will be helpful to better understand how rtfMRI NFB is working, and to identify factors that predict its clinical effects and could potentially be useful for developing personalized treatment approaches.

## Abbreviations

ACC: anterior cingulate cortex
AUD: Alcohol Use Disorder
CIMH: Central Institute of Mental Health
DBS: Deep Brain Stimulation
DSM: Diagnostic and Statistical Manual of Mental Disorders
EPI: echo-planar imaging
ICD: International Classification of Diseases
ICT: Incentive Conflict Task
NFB: neurofeedback
rIFG: right inferior frontal gyrus
rtfMRI: real-time functional magnetic resonance imaging
SKID: Structured Clinical Interview for DSM-IV
tDCS: transcranial Direct Current Stimulation
TE: echo time
TMS: transcranial magnetic stimulation
TR: time of repetition
vmPFC: ventromedial prefrontal cortex
VS: ventral striatum
